# Red Cell Distribution Width (RDW) and Complete Blood Cell Count-Derived Measures in Non-Arteritic Anterior Ischemic Optic Neuropathy

**DOI:** 10.7150/ijms.53668

**Published:** 2021-03-27

**Authors:** Antonio Pinna, Paola Carlino, Rita Serra, Francesco Boscia, Stefano Dore, Ciriaco Carru, Angelo Zinellu

**Affiliations:** 1 Department of Medical, Surgical, and Experimental Sciences, University of Sassari, Sassari, Italy.; 2 Azienda Ospedaliero-Universitaria di Sassari, Sassari, Italy.; 3 Department of Biomedical Sciences, University of Sassari, Sassari, Italy.; 4 Section of Ophthalmology, Department of Basic Medical Science, Neuroscience and Sense Organs, University of Bari, Bari, Italy.

**Keywords:** Non-arteritic Anterior Ischemic Optic Neuropathy (NA-AION), Mean Platelet Volume (MPV), Red Cell Distribution Width (RDW), Neutrophil/Lymphocyte Ratio (NLR), derived NLR (dNLR)

## Abstract

**Purpose:** To assess the role of complete blood cell count (CBC) dimensional indices and CBC-derived measures in non-arteritic anterior ischemic optic neuropathy (NA-AION).

**Methods:** In this retrospective case-control survey, 37 newly diagnosed NA-AION patients and 37 sex- and age-matched cataract controls were enrolled in 2017-2018. On the same day of NA-AION diagnosis, a blood sample was collected and CBC was determined using an automatic blood counter. CBC dimensional indices, such as mean platelet volume (MPV) and red cell distribution width (RDW), and CBC-combined indices, including neutrophil/lymphocyte ratio (NLR), derived NLR [dNLR = neutrophils/(white blood cells - neutrophils)], and platelet/lymphocyte ratio (PLR), were evaluated. Erythrocyte sedimentation rate (ESR) was also measured.

**Results:** Mean platelet count, median MPV, RDW, NLR, and dNLR were 221±48 x 10^9^/L, 8.2 fL (IQR=7.6-8.9), 13% (IQR=12-14.5), 2.50 (IQR=1.77-3.06), and 1.73 (IQR=1.31-2.07) in NA-AION patients and 248±56 x 10^9^/L, 7.60 fL (IQR=7.05-8.25), 12% (IQR=11.6-13), 1.95 (IQR=1.43-2.49) and 1.36 (IQR=1.07-1.69) in controls. NA-AION patients showed significantly lower platelet count (p=0.03) and significantly higher median values of MPV (p=0.01), RDW (p=0.015), NLR (p=0.03), and dNLR (p=0.01). Multivariate logistic regression models disclosed a significant correlation only between higher levels of RDW and NA-AION (p≤0.05). The attributable risk of the association between NA-AION and RDW was 33%.

**Conclusions:** Results suggest that RDW may be somehow involved in the pathogenesis of NA-AION. However, high-quality cohort studies are warranted to confirm whether, or not, an altered RDW may be considered a potential biomarker of this vascular disorder affecting the optic nerve.

## Introduction

Non-arteritic anterior ischemic optic neuropathy (NA-AION) is the most frequent form of acute optic neuropathy in individuals aged over 50 years, affecting between 2.3 and 10.2 people per 100,000 population annually in the U.S. and accounting for up to 6,000 new cases every year 1, 2.

NA-AION is a multifactorial disease. Systemic hypertension, diabetes mellitus, atherosclerosis, and a crowded optic disc are well-known risk factors 3, 4. Former reports have suggested that systemic and local inflammation may affect the pathogenesis of NA-AION, by inducing both atherosclerosis and hypercoagulability 5-11. Inflammation is the result of complex interactions between the immune and inflammatory system, which includes neutrophils, macrophages, and lymphocytes. The absolute counts of white blood cells (WBC), neutrophils, and lymphocytes, and their ratios can be reliable indicators of chronic inflammation 12. Neutrophil/lymphocyte ratio (NLR) has become an important biomarker of systemic inflammation and an independent prognostic factor in some chronic diseases and solid malignancies 12. Furthermore, recent studies have reported higher NLR values in NA-AION patients 10, 13, 14.

The role of platelets is crucial in thrombo-occlusive disorders, such as ischemic and hemorrhagic stroke 15 and retinal vein occlusion (RVO) 16, 17. Recent studies have suggested that platelets may also been implicated in the pathogenesis of NA-AION 14, 18. Mean platelet volume (MPV) is an important marker of the production rate and size of platelets and has been correlated to platelet activation 19. Larger platelets are metabolically more reactive and aggregate more easily.

The purpose of this study was to evaluate the role of complete blood cell count (CBC) dimensional measures, such as MPV and red cell distribution width (RDW), and CBC-combined indices, including NLR, derived NLR [dNLR = neutrophils/(white blood cells - neutrophils)], and platelet/lymphocyte ratio (PLR), in NA-AION.

## Methods

This was a retrospective case-control study, including 37 newly diagnosed NA-AION patients and 37 sex- and age-matched subjects with no evidence of NA-AION, examined at the Ophthalmology Unit, Department of Medical, Surgical and Experimental Sciences, University of Sassari, Sassari, Sardinia, Insular Italy, in 2017-2018. To ensure appropriate matching, patients of non-Sardinian descent were excluded.

Ethical approval was waived by the local Ethics Committee of the Azienda Ospedaliero-Universitaria di Sassari, Sassari, Italy, in view of the retrospective nature of the survey, which was conducted in compliance with the tenets of the Declaration of Helsinki. Each participant received detailed information and provided informed consent before inclusion.

The inclusion criterion for the case group was the diagnosis of NA-AION. This diagnosis was established by the history of sudden visual loss together with optic disc swelling, characteristic visual field defects, and typical fluorescein angiography features. The time span for considering the patients as “newly” diagnosed was ten days. All NA-AION patients underwent a complete eye examination, consisting of best corrected visual acuity (BCVA), slit-lamp evaluation, applanation tomometry, fundoscopy, fluorescein angiography, and automated visual field examination (Humphrey Field Analyzer 30-2 test). Visual field defects were classified by a team of readers (A.P. and P.C.). Medical conditions, including diabetes mellitus, systemic hypertension, increased blood cholesterol levels, cardio-vascular status (presence of angina and/or myocardial infarction), and cerebro-vascular status (presence of transient ischemic attacks and/or stroke) were recorded. Exclusion criteria included non-Sardinian descent and evidence of any other neurological, systemic, or ophthalmic disorder that could account for optic disc swelling and visual loss.

One sex- and aged-matched control per each case was randomly selected from the cataract registry. Subjects with present or past history of AION, RVO, or retinal artery occlusion were excluded. All controls underwent a complete eye examination, consisting of BCVA, slit-lamp biomicroscopy, applanation tonometry, and dilated fundus evaluation. The same medical conditions as for NA-AION patients were also recorded.

Definitions of systemic hypertension, diabetes, and hypercholesterolemia have been reported previously 20.

On the same day of NA-AION diagnosis, blood samples were collected by standard venipuncture, and blood cell counts were made using an automatic blood counter Cell-Dyn Sapphire (Abbott Diagnostics, Santa Clara, CA, USA) in a certified laboratory. Erythrocyte sedimentation rate (ESR) was also measured using standard techniques. Blood sample collection for routine tests is standard procedure for all AION and cataract patients.

Blood cell counts, dimensional indices (MPV, RDW), and combined indices, such as NLR, dNLR, and PLR, were evaluated.

Results were reported as mean ± standard deviation (SD) or median and interquartile range (IQR) values, as appropriate. Shapiro-Wilk test was used to assess variable distribution. Student's t-test, Welch-test for data with unequal variances, or Mann-Whitney rank sum test was used to assess statistical differences between groups.

The ability of platelet number, MPV, RDW, NLR, and dNLR to predict NA-AION was determined using receiver operating characteristics (ROC) curve analysis. The optimal cut-off for sensitivity and specificity was calculated by maximizing the Youden's index.

Multivariate logistic regression models, including all covariates found to be significantly associated with NA-ION in univariate logistic regression analysis (i.e., platelet number, MPV, RDW, NLR, and dNLR) were used. NLR and dNLR were assessed separately to avoid collinearity. Odds ratios (ORs) and 95% confidence intervals (CIs) were determined.

P values ≤0.05 were considered to be statistically significant. Statistical analysis was performed using MedCalc for Windows, version 15.4 64 bit (MedCalc Software, Ostend, Belgium).

The attributable risk of the association between NA-AION and RDW was calculated. For this purpose, the upper limit of the reference range for RDW (14.5%) was used.

## Results

One percent of the patients with NA-AION and 1% of the controls eligible for this retrospective survey declined to participate. The main reason was “not interested”.

The study group included 37 newly diagnosed NA-AION patients (23 men, 14 women; mean age: 65.6±12.4 years). No patient reported pain with eye movements before visual loss. All eyes with NA-AION had no vision improvement over time. Automated perimetry revealed an altitudinal defect in 22 (60%) eyes and generalized depression in 15 (40%). All affected eyes had extensive leakage of fluorescein in the swollen portion of the optic disc.

Demographics, medical history details, and CBC results of NA-AION patients and cataract controls with no evidence of NA-AION are presented in Table 1. NA-AION patients and controls had similar percentages of systemic hypertension and hypercholesterolemia. Similarly, there were no significant differences in WBC, lymphocyte, and neutrophil counts and ESR values. Conversely, NA-AION patients showed significantly lower mean values of platelet number, significantly higher rates of diabetes mellitus and cerebro/cardiovascular disease, and significantly higher median values of MPV, RDW, NLR and dNLR. These values were acutely altered around the time of NA-AION onset.

Table 2 shows ROC curve analysis results for platelet count, MPV, RDW, NLR, and dNLR. The area under the curve (AUC) values disclosed a good specificity only for dNLR, whereas all the indices analyzed showed a relatively poor sensitivity.

Results of multivariate logistic regression models, including platelet number, MPV, RDW, and NLR or dNLR as covariates are presented in Table 3. A statistically significant relationship between RDW and NA-AION (p≤0.05) was found.

RDW accounted for 33% of the attributable risk for NA-AION.

## Discussion

NA-AION is a leading cause of acute loss of vision in individuals aged 55 to 70 years. This condition is thought to be the result of circulatory failure within the optic disc. Many systemic disorders, which may reduce optic disc perfusion, have been reported as risk factors. Among these there are systemic hypertension 3, 4, 21, diabetes mellitus 3, 4, 22, hypercholesterolemia 4, 23, ischemic heart disease 3, acute blood loss 24, and marked nocturnal arterial hypotension, especially in patients taking anti-hypertensive medications 25. Furthermore, a crowded optic disc may be a predisposing factor for NA-AION, either by mechanically impairing the axoplasmic flow or amplifying the microvascular compression secondary to optic disc swelling of any cause 26. On the other hand, Glucose-6-Phosphate Dehydrogenase (G6PD) deficiency may have a protective effect against the development of NA-AION 27.

The exact pathophysiological mechanisms underlying NA-AION are still unclear. The rapid onset, stable course with no or minimal visual improvement, and association with circulatory risk factors are suggestive of a vascular origin. According to the most reliable theory, impairment of the optic disc circulation, aggravated by axonal *crowding*, eventually reaches a point at which insufficient oxygen levels cause ischemia and disc edema 28. A vicious circle of ischemia, swelling of axons, microvascular compression, and further ischemia leads to progressive optic nerve head damage. Nocturnal arterial hypotension contributes to the worsening of ischemia 25.

Platelets play a critical role in thrombo-occlusive disease. MPV is an important marker of platelet size and activity. Larger platelets produce more thromboxane A2, express more glycoprotein Ib and glycoprotein IIb/IIIa receptors, and aggregate more easily 19, 29.

In our survey, patients with NA-AION showed a significantly lower platelet count and significantly larger platelets than controls. Former investigations have reported a correlation between NA-AION and increased MPV 11, 13, 14, 18, a result consistent with our study. Furthermore, Stiebel-Kalish et al. 30, in a systematic review and meta-analysis on aspirin use after NA-AION, concluded that there is weak evidence showing a beneficial effect of acetylsalicylic acid in preventing NA-AION in the second eye. On the whole, these findings support the idea that platelets may be somehow involved in the pathogenesis of NA-AION. However, while platelets do play an important role in thrombo-occlusive disease, their role in NA-AION is less clear. According to Hayreh 31, in the typical NA-AION, which is a hypotensive disorder, there is no scientific rationale for aspirin use in preventing recurrences of NA-AION, if not for a small number of cases due to true embolism, where it may be beneficial 31.

Changes in CBC dimensional indices, such as MPV and RDW, reflect inflammation severity in several disorders, because systemic inflammation not only affects the survival of platelets and red blood cells, but also deforms their membranes 32. RDW is an index measuring variation in red blood cell size or volume (anisocytosis). Generally, it is expressed as the ratio between the standard deviation of erythrocyte volume and mean corpuscular volume (MCV), multiplied by 100. RDW has historically been used to help classify anemia. However, it has recently been implicated in subclinical inflammation, as well as in other human diseases different from anemia, including chronic heart failure, myocardial infarction, stroke, liver impairment, pregnancy complications, and NA-AION 14, 33, 34. In our investigation, we observed that RDW values were higher in NA-AION patients than in controls.

NLR is a broadly accessible, easy to calculate, and cheap inflammatory biomarker. Clinical evidence suggests that it may predict disease development, progression, and prognosis and response to treatment. A relationship between NLR and disease activity and outcome has been observed in several malignancies and chronic inflammatory disorders, including arthritis, diabetes mellitus, systemic hypertension, and chronic obstructive pulmonary disease 12, 35-38. An association between the severity of diabetic retinopathy and NLR has been documented 39, whereas contrasting data have been reported about the role of NLR in AMD 40-42.

In this study, patients with newly diagnosed NA-AION had significantly higher NLR and dNLR values than cataract controls with no evidence of present or past NA-AION. Likewise, in a retrospective study on 45 NA-AION patients and 50 age- and sex-matched controls, Polat et al. 13 found that NA-AION patients had significantly higher values of NLR. A similar result was reported by Gunes et al. 10 in a survey comparing 56 NA-AION patients with 56 healthy subjects. These authors also found that ROC curves for NLR predicted NA-AION development with a sensitivity of 85% and specificity of 41% 10. Conversely, Inanc et al. 11 observed no correlation between NLR and NA-AION.

In our study, dNLR showed a higher statistical significance than NLR (p=0.01 vs p=0.03). Furthermore, AUC values disclosed a higher specificity for dNLR (81% vs 68%). Overall, these findings seem to support the possibility that dNLR might be a more reliable biomarker than NLR in patients with NA-AION.

Multivariate logistic regression models disclosed a significant correlation only between increased RDW and NA-AION (p≤0.05). The attributable risk of the association between NA-AION and RDW was 33%. The clinical meaning of these findings is not clear. Recently, Ozkok et al. 43 have reported that RDW was significantly higher and correlated significantly with baseline and final visual acuity in RVO patients. These authors postulated that RDW may represent an integrative measure of a multifactorial disease process, involving oxidative stress, chronic inflammation, and endothelial dysfunction, which contribute to RVO pathogenesis. Possibly, this may also be the case in NA-AION.

Our study has some intrinsic limitations, such as the retrospective nature and case-control design. This type of design usually do not allow to determine the temporal relationship between exposure and disease; therefore, it cannot be clearly determined whether the higher values of MPV, RDW, NLR, and dNLR are possible causes of NA-AION or simply correlates of other factors associated with NA-AION. Prospective studies may provide a more reliable evidence to look for a potential cause-effect relationship between these dimensional and CBC-combined indices and NA-AION. Another limitation of this study is the fact that the cases and controls were not matched for certain parameters which might have affected CBC indices, such as body mass index, diabetic control, anemia, anti-hypertensive medications, and smoking history. Furthermore, we do not know how many patients had a crowded optic disc, a known risk factor for NA-AION, because data about optic disc size were incomplete. Last, but not least, our survey was performed on a relatively small, genetically homogeneous group of subjects of Sardinian ancestry. Consequently, our results might not be extended to NA-AION patients of non-Sardinian descent.

To sum up, our results suggest that RDW may be a potential disease biomarker in patients with NA-AION. However, high-quality cohort studies are warranted before any definitive conclusion on the putative role of increased RDW on NA-AION can be drawn.

## Figures and Tables

**Table 1:** Demographic data, medical history details, and blood cell count results of patients with non-arteritic anterior ischemic optic neuropathy (NA-AION) and cataract controls without NA-AION

	NA-AION patients (37)	Controls (37)	p-value
Age, years, mean ± Standard Deviation (SD)	65.6±12.4	65.6±12.4	0.99
Gender (males/females)	23/14	23/14	1
Diabetes, n (%)	5 (13.5)	1 (2.7)	0.09
Hypertension, n (%)	17 (46.3)	17 (46.3)	1.00
Hypercholesterolemia, n (%)	10 (27.0)	12 (32.4)	0.61
Cerebro-/Cardio-vascular disease, n (%)	9 (24.3)	3 (8.11)	0.06
White Blood cell Count, × 10^9^/L, median (IQR)	6.60 (5.93-8.33)	6.67 (6.03-8.44)	0.82
Lymphocytes, × 10^9^/L, mean ± SD	1.88±0.55	2.14 ±0.67	0.24
Neutrophils, × 10^9^/L, median (IQR)	4.00 (3.28-5.15)	3.80 (3.35-5.05)	0.53
Platelet number, × 10^9^/L, mean ± SD	221±48	248±56	0.03
Mean Platelet Volume [MPV] (fl), median (IQR)	8.20 (7.60-8.90)	7.60 (7.05-8.25)	0.013
Red cell Distribution Width [RDW] (%), median (IQR)	13.0 (12.0-14.5)	12.0 (11.6-13.0)	0.015
Neutrophil/Lymphocyte Ratio (NLR), median (IQR)	2.50 (1.77-3.06)	1.95 (1.43-2.49)	0.03
Derived NLR (dNLR), median (IQR)	1.73 (1.31-2.07)	1.36 (1.07-1.69)	0.01
Platelet/Lymphocyte Ratio (PLR), median (IQR)	116 (98-147)	117 (97-152)	0.97
Erythrocyte sedimentation rate (mm/hr), mean ± SD	22.5 ± 16.4	19.9 ± 14.8	0.48

**Table 2:** Receiver operating characteristics (ROC) curves and prognostic accuracy of platelet number, mean platelet volume (MPV), red cell distribution width (RDW), neutrophil/lymphocyte ratio (NLR), and derived NLR (dNLR) in non-arteritic anterior ischemic optic neuropathy

Marker	AUC	95%CI	p value	Cut-off	Sensitivity	Specificity
Platelet number	0.657	0.537-0.763	0.016	<240	70.27	62.16
MPV	0.669	0.549-0.775	0.008	>7.8	62.16	63.89
RDW	0.664	0.544-0.769	0.009	>12	70.27	59.46
NLR	0.645	0.526-0.753	0.026	>2.11	62.16	67.57
dNLR	0.671	0.552-0.776	0.008	>1.72	54.05	81.08

**Table 3:** Multivariate logistic regression analysis models, showing odds ratios for non-arteritic anterior ischemic optic neuropathy (NA-AION = 37)

	Model with NLR			Model with dNLR		
	OR	95% Confidence Interval	p value	OR	95% Confidence Interval	p value
RDW	1.453	0.991-2.131	0.05	1.474	1.0001-2.173	0.049
Platelets	0.996	0.985-1.007	0.44	0.996	0.985-1.007	0.48
MPV	1.354	0.814-2.252	0.24	1.375	0.821-2.302	0.23
NLR	1.604	0.873-2.948	0.13	--	--	--
dNLR	--	--	--	2.63	0.958-7.220	0.061

Number of controls: 37. RDW: red cell distribution width; MPV: mean platelet volume; NLR: neutrophil/lymphocyte ratio; dNLR: derived NLR.
